# A PDE10A inhibitor CPL500036 is a novel agent modulating striatal function devoid of most neuroleptic side-effects

**DOI:** 10.3389/fphar.2022.999685

**Published:** 2022-11-09

**Authors:** Mikolaj Matloka, Sylwia Janowska, Piotr Pankiewicz, Sofiya Kokhanovska, Tomasz Kos, Małgorzata Hołuj, Izabela Rutkowska-Wlodarczyk, Krzysztof Abramski, Monika Janicka, Piotr Jakubowski, Maciej Świątkiewicz, Marlena Welniak-Kaminska, Joanna Hucz-Kalitowska, Paulina Dera, Lukasz Bojarski, Paweł Grieb, Piotr Popik, Maciej Wieczorek, Jerzy Pieczykolan

**Affiliations:** ^1^ R&D Centre, Celon Pharma SA, Kazuń Nowy, Poland; ^2^ Department of Behavioral Neuroscience and Drug Development, Maj Institute of Pharmacology, Polish Academy of Sciences, Kraków, Poland; ^3^ Mossakowski Medical Research Institute, Polish Academy of Sciences, Warsaw, Poland

**Keywords:** CPL500036, PDE10A inhibitor, lead characterization, drug development, safety evaluation

## Abstract

**Background:** Phosphodiesterase 10A (PDE10A) is expressed almost exclusively in the striatum and its inhibition is suggested to offer potential treatment in disorders associated with basal ganglia. We evaluated the selectivity, cytotoxicity, genotoxicity, pharmacokinetics and potential adverse effects of a novel PDE10A inhibitor, CPL500036, *in vivo*.

**Methods:** The potency of CPL500036 was demonstrated by microfluidic technology, and selectivity was investigated in a radioligand binding assay against 44 targets. Cardiotoxicity *in vitro* was evaluated in human ether-a-go-go related gene (hERG)-potassium channel-overexpressing cells by the patch-clamp method and by assessing key parameters in 3D cardiac spheroids. Cytotoxicity was determined in H1299, HepG2 and SH-SY5Y cell lines. The Ames test was used for genotoxicity analyses. During *in vivo* studies, CPL500036 was administered by oral gavage. CPL500036 exposure were determined by liquid chromatography–tandem mass spectrometry and plasma protein binding was assessed. The bar test was employed to assess catalepsy. Prolactin and glucose levels in rat blood were measured by ELISAs and glucometers, respectively. Cardiovascular safety *in vivo* was investigated in dogs using a telemetry method.

**Results:** CPL500036 inhibited PDE10A at an IC_50_ of 1 nM, and interacted only with the muscarinic M2 receptor as a negative allosteric modulator with an IC_50_ of 9.2 µM. Despite inhibiting hERG tail current at an IC_25_ of 3.2 μM, cardiovascular adverse effects were not observed in human cardiac 3D spheroids or *in vivo*. Cytotoxicity *in vitro* was observed only at > 60 μM and genotoxicity was not recorded during the Ames test. CPL500036 presented good bioavailability and penetration into the brain. CPL500036 elicited catalepsy at 0.6 mg/kg, but hyperprolactinemia or hyperglycemic effects were not observed in doses up to 3 mg/kg.

**Conclusion:** CPL500036 is a potent, selective and orally bioavailable PDE10A inhibitor with a good safety profile distinct from marketed antipsychotics. CPL500036 may be a compelling drug candidate.

## 1 Introduction

Schizophrenia is a highly debilitating psychiatric disorder characterized by a broad spectrum of symptoms grouped into “positive”, “negative”, and “cognitive” clusters. Disease-modifying treatments have not been developed because schizophrenia pathophysiology is complex and incompletely understood (Millan et al., 2016).

Symptomatic therapy includes first- and second-generation antipsychotic agents. They treat the positive symptoms (e.g., delusions, hallucinations) of schizophrenia, but their efficacy against the negative symptoms (e.g., diminished expression and avolition) and cognitive deficits (e.g., impaired performance of verbal and working memory and executive functions) is very limited (McCutcheon et al., 2020). Moreover, resistance to therapies develops often, and results in no response to treatment in 20%–30% and in a moderate response in 30% of patients suffering from schizophrenia (Steeds et al., 2015). Side-effects remain another challenge. First-generation antipsychotics such as haloperidol and chlorpromazine are highly selective against dopamine type 2 (D2) receptors and act as full antagonists. Highly effective blockade of D2 receptors in basal ganglia circuits is associated with acute extrapyramidal symptoms (EPS) such as catalepsy, and receptor blockade in the pituitary gland results in hyperprolactinemia, which can lead to gynecomastia or amenorrhea (Huhn et al., 2019).

In search of medication free of the aforementioned effects, a second generation of antipsychotics was developed. Multimodal affinity maintained the efficacy of second-generation antipsychotics in reducing EPS and hyperprolactinemia. However, metabolic syndrome is the main concern (De Hert et al., 2012). Metabolic syndrome includes glucose intolerance that results in diabetes mellitus, weight gain and dyslipidemia (Pillinger et al., 2020). One of the most severe side-effects caused by antipsychotics of both generations are adverse cardiovascular effects (De Hert et al., 2012). Prolongation of the QT interval resulting in Torsades de Pointes that may lead to a sudden death is a consequence of using certain antipsychotics (Haddad and Anderson, 2002). Moreover, cardiotoxicity such as cardiomyopathy, myocarditis and pericarditis has been observed, in particular for clozapine (Krebs et al., 2006). The latter and other antipsychotics can also cause leukopenia or agranulocytosis that leads to changes in psychosis management (Flanagan and Dunk, 2008). All side-effects are a substantial factor of poor compliance or non-compliance of patients leading to ∼50% full adherence to treatment (Perkins, 2002). Together with drug resistance and poor management of negative symptoms and cognitive symptoms, a constant need for medication with a new mechanism of action is a rational approach for development of drugs against schizophrenia.

CPL500036 {7-[5,8-Dimethyl-(1,2,4) triazolo (1,5-a) pyrazin-2-yl]-2-phenylimidazo (1,2-a) pyrimidine} is a novel phosphodiesterase 10A (PDE10A) inhibitor. It has high potency and selectivity against PDE10A compared with those of other phosphodiesterases (Moszczyński-Pętkowski et al., 2018). PDE10A is a cyclic adenosine monophosphate (cAMP)- and cyclic guanosine monophosphate-hydrolyzing enzyme. It is highly enriched in striatal medium spiny neurons expressing D1 (direct pathway) and D2 dopamine receptors (indirect pathway) (Wang et al., 2007). Inhibition of PDE10A expression leads to modulation of cyclic-nucleotide levels in indirect and direct pathways of basal-ganglia circuits to result in disinhibition and activation of those pathways, respectively. It has been postulated that PDE10A inhibition may be a promising strategy for several disorders connected to disturbed neurotransmission of striatal and basal ganglia, including schizophrenia, Huntington’s disease, Parkinson’s disease or stroke (Giampà et al., 2010; Jankowska et al., 2018; Birjandi et al., 2020; Lenda et al., 2021).

We aimed to evaluate the selectivity against common and pharmacologically relevant off-targets, cytotoxicity, genotoxicity, pharmacokinetics and potential adverse effects of CPL500036 (including catalepsy, metabolic syndrome, hyperprolactinemia, and cardiotoxicity). CPL500036 is under a phase-2 clinical trial as treatment for schizophrenia and levodopa-induced dyskinesia in Parkinson’s disease (NCT05297201, NCT05278156).

## 2 Materials and methods

### 2.1 Drugs

CPL500036 was synthesized by our research team. It was used as a hydrochloride salt in all experiments except for *in vivo* cardiovascular assessment (where a free-base form of the compound was applied). For *in vivo* studies, CPL500036 was suspended in 0.5% methylcellulose solution (MilliporeSigma, Burlington, MA, United States) and 2% Tween80 (MilliporeSigma) for oral administration at 5 ml/kg, or in 5% N-Methyl-2-pyrrolidone (MilliporeSigma)/30% polyethylene glycol-300 (Merck, Whitehouse Station, NJ, United States) saline solution for intravenous administration at 2 ml/kg. Haloperidol (VWR, Darmstadt, Germany) was suspended in 0.5% methylcellulose solution and 2% Tween80. Olanzapine (Neuland Laboratories, Hyderabad, India) was dissolved in minimal amounts of 0.1 N HCl and diluted with saline.

### 2.2 Determination of the half-maximal inhibitory concentration (IC_50_) against PDE10A

IC_50_ determination was assessed using LabChip^®^ microfluid technology (PerkinElmer, Hopkinton, MA, United States) in line with the service-provider’s protocol. CPL500036 was tested at eight concentrations ranging from 0.1 nM to 300 nM. The assay was run on the EZ Reader^™^ II platform (Caliper Life Sciences, Hopkinton, MA, United States). After the incubation period, levels of the reaction products and remaining substrate were measured. Analyzed data were from duplicate measurement, and IC_50_ was determined using a variable slope with Prism 5 (GraphPad, San Diego, CA, United States).

### 2.3 Selectivity

The SafetyScreen44^®^ assay was carried out by Eurofins (Poitiers, France) according to the service-provider’s protocol. CPL500036 (1 µM) was tested against a panel of 47 potential off-targets proteins, including G protein-coupled receptors (GPCRs), transporters, ion channels, nuclear receptors kinases and other non-kinase enzymes, by use of a set of the binding assays.

Binding of CPL500036 was calculated as the percent binding inhibition of a radioactively labeled ligand specific for each target. Enzyme inhibition of CPL500036 was calculated as the percent inhibition of the activity of the control enzyme. “No interaction” was denoted if inhibition ≤ 25% of specific binding of the control. Determination of muscarinic acetylcholine receptor (M2) activity induced by CPL500036 (antagonism and agonism) was undertaken in human recombinant CHO cells by measurement of the intracellular cAMP level according to the service-provider’s protocol. IC_50_ (concentration causing a half-maximal inhibition of specific binding of the control) or EC_50_ (concentration causing a half-maximal response of specific binding of the control) and the Hill coefficient were determined by non-linear regression analysis of the competition curves generated with mean replicate values using Hill-equation curve fitting with software developed at Cerep (Celle-Lévescault, France).

### 2.4 Inhibition of potassium voltage-gated channel subfamily H member 2

The dose-dependent effect of CPL500036 (10^–7^ M–10^–5^ M) on hERG current was conducted at Centre De Recherches Biologiques (CERB; Baugy, France) using the whole hERG-HEK-293 transfected cell system and manual patch-clamp method. The study was performed in compliance with the Good Laboratory Practice (GLP). Cells were clamped to −80 mV, depolarized to 0 mV for 5 s (to allow activation of hERG current) and repolarized to −50 mV for 5 s (to allow hERG tail current to deactivate). This experimental procedure was repeated at a frequency of 0.06 Hz. Currents were filtered at 1 kHz and acquired at a frequency of 2 kHz. The amplitude of hERG tail current was measured during the repolarizing pulse from 0 mV to −50 mV. Cells were perfused with Tyrode’s solution (CERB), the vehicle and subsequently with Tyrode’s solution containing CPL500036 for 5 min until steady state was reached for each perfusion period. Currents were measured before and after exposure to CPL500036.

### 2.5 Three-dimensional cardiac spheres

The cardiotoxic effect of CPL500036 was evaluated in spontaneously beating cardiac 3D spheroids (comprising cardiomyocytes and fibroblasts derived from human pluripotent cells) by Cyprotex (Cheshire, United Kingdom). Experiments were carried out in accordance with the service-provider’s protocol. Specific parameters of cell health were assessed: number and size of spheroids, DNA structure, intracellular level of calcium ions, mitochondrial mass, membrane potential and cellular ATP. CPL500036 was tested at 0.0457–100 µM (*n* = 2). During the 14 days of the experiment, redosing occurred on three occasions. Spheroid hypertrophy was measured on days 3, 7, 10, and 14 using the “brightfield live cellular imaging” mode of a Cellomics ArrayScan^®^ XTI system (Thermo Scientific, Waltham, MA, United States). On day-14, the spheroid model was analyzed using the confocal mode of Cellomics ArrayScan XTI following incorporation of appropriate fluorescent dyes. Subsequently, cellular ATP content was measured using CellTiterGlo^®^ (Promega, Fitchburg, WI, United States). Mitomicyn C and dasatinib were used as positive controls (Supplementary Table S1).

### 2.6 Cytotoxicity

The cytotoxicity of CPL500036 was evaluated in human non-small-cell lung cancer (NCI-H1299, ATCC^®^ CRL-5803^™^), human hepatocellular carcinoma (Hep G2 [HEPG2], ATCC^®^ HB-8065^™^) and human neuroblastoma (SH-SY5Y, ECACC, 94030304) cells using the 3-(4,5-dimethylthiazol-2-yl)-2,5-diphenyltetrazolium bromide (MTT) assay (Invitrogen, Carlsbad, CA, United States). Cells were seeded in an appropriate complete growth medium (following manufacturer recommendations) onto 96-well plates. After overnight incubation (37°C, 5% CO_2_) the medium was replaced with fresh medium (100 μl/well) containing the desired concentration of CPL500036 (8 nM–60 μM, diluent factor = 3), positive control or vehicle control (1% dimethyl sulfoxide (DMSO)) followed by incubation for 72 h (37°C, 5% CO_2_). Then, 25 μl of filtered (filter size = 0.22 μm) MTT solution (12 mM) in phosphate-buffered saline (PBS) was added to each well, including blank control (medium without cells) followed by incubation for 2 h (37°C, 5% CO_2_). After that, formazan crystals were dissolved in DMSO (100 μl/well). The absorbance was measured at 540 nm (A540, substrate wavelength) and 690 nm (A690, reference wavelength) using an automatic microplate reader (Multiskan^™^ GO; ThermoFisher Scientific). The metabolic activity of cells in the presence of vehicle control (1% DMSO) was considered to be 100%, and we calculated the difference between A540 and A690. IC_50_ was calculated using Prism 7.05. Results on positive controls are included in to the supplementary data (Supplementary Figure S2).

### 2.7 Genotoxicity

The study of CPL500036-induced genotoxicity was conducted by TOXI-COOP ZRT (Budapest, Hungary) according to the service-provider’s protocol and in compliance with the Good Laboratory Practice (GLP). The compound was tested at a concentration range of 0.025 mg/ml–25 mg/ml. Mutagenicity was evaluated in histidine-requiring auxotroph strains of *Salmonella typhimurium* (TA98, TA100, TA1535, and TA1537) and the tryptophan-requiring auxotroph strain of *Escherichia coli* (WP2 uvrA) in the presence and absence of a post-mitochondrial supernatant (S9) prepared from the livers of phenobarbital/β-naphthoflavone-induced rats. Results on positive controls are included in to the supplementary data (Supplementary Table S2).

### 2.8 Plasma protein binding

The plasma protein binding study was carried out by Eurofins (Poitiers, France) according to the service-provider’s protocol. Briefly, the protein matrix was spiked with the test compound at 10 μM (*n* = 2) with a final DMSO concentration of 1%. The dialysate compartment was loaded with phosphate buffered saline (PBS, pH 7.4) and the sample side was loaded with equal volume of the spiked protein matrix. The dialysis plate was incubated at 37°C for 4 h. After the incubation, samples were taken from each compartment, diluted with the phosphate buffer followed by addition of acetonitrile and centrifugation. The supernatants were then used for HPLC-MS/MS analysis. Acebutolol, quinidine, and warfarin were tested as reference.

### 2.9 Pharmacokinetics

Procedures on rats were undertaken according to guidelines set by the Polish Ethical Committee on Animal Researchm and approved by the Local Ethics Committee for Animal Experiments in Gdańsk, Poland. Procedures on dogs were in compliance to the European convention for the protection of vertebrate animals used for experimental and other scientific purposes (ETS 123), the Czech Collection of laws No. 246/1992, inclusive of the amendments, on the Protection of animals against cruelty, Public Notice of the Ministry of Agriculture of the Czech Republic, Collection of laws No. 419/2012 as amended, on keeping and exploitation of experimental animals and approved by the Institutional Animal Care and Use Committee (IACUC) and the Committee for Animal Protection of the Ministry of Health of the Czech Republic (08/2016).

The study was carried out in 40 male Wistar Han rats (∼10 weeks; Tri-City Academic Laboratory Animal Centre, Research and Services Centre, Medical University of Gdansk). Rats were fasted for 12 h (water was available *at libitum*) before administration and fed 4 h post-dose. Rats received CPL500036 (3 mg/kg per oral gavage). At each of eight time points (15 min, 30 min as well as 1 h, 2 h, 4 h, 7 h, 12 h, and 24 h), five rats were killed by exsanguination, and samples of blood and brain were harvested. Two male and two female Beagle dogs (10.5–13 months old, MediTox s.r.o., Konarovice, Czech Republic) received CPL500036 in a dose of 1.5 mg/kg per oral gavage. Blood was taken by venepuncture from *v. cephalica antebrachii* at twelve time points (0 min, 15 min, 30 min as well as 1 h, 2 h, 3 h, 4 h, 6 h, 8 h, 12 h, 24 h, and 48 h.

Blood was collected into tubes containing the anticoagulant K2EDTA and centrifuged (2,000 × *g*, 15 min, 4°C) to obtain plasma. Brain samples were homogenized in water. Plasma and brain homogenates were stored at −80°C until analyses. Acetonitrile (150 μl) spiked with internal standard (donepezil; MilliporeSigma) was added to 50 μl of each plasma homogenate or brain homogenate, respectively. The mixture was shaken vigorously for 2 min and then centrifuged (4,000 × *g*, 4 min, room temperature). Supernatants were injected into a high-performance liquid chromatography system (Infinity^™^ 1,260; Agilent Technologies, Santa Clara, CA, United States) with a Zorbax SB column (50 cm × 2.1 cm; 1.8 μm; Agilent Technologies). The mobile phase was ammonium formate (20 mM, pH 3.0) and acetonitrile with 0.1% formic acid. The gradient chromatographic separation used a flow rate of 0.4 ml/min for 7.5 min at a nominal temperature of 30°C. Signal acquisition was undertaken for 5.5 min using of a triple quadrupole mass spectrometer (6,460 series; Agilent Technologies) with electrospray ionization (5500 V, 320°C). Multiple-reaction monitoring was applied to detect the following transitions of the *m/z* ratio: 342.0–220.9 and 342–235.9 for CPL500036; 380.5–362.9 and 380.5–243.5 for donepezil. The drug concentration was calculated from the ratio of the peak area response relative to the internal standard using a standard curve.

### 2.10 Catalepsy

The catalepsy test was conducted in accordance with the *Guide for the Care and Use of Laboratory Animals* (United States National Institutes of Health, Bethesda, MD, United States) and approved by the II Local Ethics Committee for Animal Experiments at the Maj Institute of Pharmacology (Polish Academy of Sciences, Krakow, Poland).

Sprague–Dawley rats (∼300 g; Charles River Laboratories, Wilmington, MA, United States) (*n* = 8–12/group) were treated (p.o.) with vehicle or CPL500036 (0.15 mg/kg, 0.3 mg/kg, 0.6 mg/kg or 2 mg/kg). As a positive control causing catalepsy, haloperidol (1 mg/kg, i.p.) was employed. One hour following drug administration, catalepsy (prolonged maintenance of an externally imposed abnormal posture) was assessed using the bar test as described by Ionova and Severtsev (Ionov and Severtsev, 2012). Testing was accomplished by placing each rat in an upright position with his forepaws resting on a horizontal bar (0.9 cm in diameter) suspended in a wooden frame 7.5 cm above the cage floor. The rat was allowed to keep his forepaws on the bar for a measurement lasting for 30 s. The latency to remove both forepaws from the bar was measured with a hand-held stopwatch. The test was repeated for each rat every 30 min up to 240 min. Rats were returned to their home cages between tests. Each rat was tested twice, at an interval of 1 week.

### 2.11 Hyperprolactinemia

Animal procedures were carried out according to guidelines set by the Polish Ethical Committee on Animal Research, and approved by the Local Ethics Committee for Animal Experiments in Warsaw, Poland.

Four male Sprague–Dawley rats (∼10 weeks; Mossakowski Medical Research Institute, Polish Academy of Sciences, Warsaw, Poland), underwent gavage with CPL500036 (3 mg/kg), haloperidol (3 mg/kg; as a positive control) or vehicle. Rats were fasted for 16 h before administration. Blood samples from the tail vein were collected immediately before and 2 h after administration. Plasma was obtained by centrifugation (1,600 × g, 20 min, 4°C) of blood samples collected on K2EDTA, and was stored at −80°C until analyses. The prolactin concentration in plasma samples was determined using the Prolactin (rat) EIA kit (Bertin Rockville, MD, United States) according to the manufacturer’s protocol. The prolactin concentration difference was assessed as follows:

$$\%∆prolactin=\frac{c_{prolactin,\;2h}-c_{prolactin,\;0h}}{c_{prolactin,\;2h}}*100\%$$ (1)

### 2.12 Glucose tolerance test

Animal procedures were undertaken according to the guidelines of the Polish Ethical Committee on Animal Research, and approved by the Local Ethics Committee for Animal Experiments in Gdańsk, Poland.

Thirty-two male Wistar Han rats (∼10 weeks; Tri-City Academic Laboratory Animal Centre-Research and Services Centre, Medical University of Gdansk) were fasted for 16 h before experimentation. Animals underwent gavage with CPL500036 (0.3 mg/kg, 1 mg/kg or 3 mg/kg) or vehicle 2 h before receiving a glucose bolus (2 g/kg, i.p.; MilliporeSigma). Olanzapine (10 mg/kg) was used as an active comparator and delivered (s.c.) 30 min before the glucose bolus. Blood samples from the tail vein were collected from each rat right before drug administration, just before glucose loading as well as 15 min, 30 min, 60 min, 120 min, and 180 min after glucose administration. The glucose concentration was measured using a glucometer (Accu-ChekGo, Roche Diagnostics, Mannheim, Germany).

### 2.13 *In vivo* cardiovascular safety

The animal housing and procedures used were in compliance with Spanish Law RD 53/2013 and European Directive 2010/63/UE (Legislation for the protection of animals used for scientific purposes; Estado). Animal procedures were approved by Committee on Ethics in animal experimentation Vivotecnia (Madrid, Spain). The study was performed in compliance with the Good Laboratory Practice (GLP).

Four male Beagle dogs who had been implanted previously with telemetry transmitter devices (Data Sciences, Saint Paul, MN, United States) were used. The number of animals corresponds to the standard approach used for such studies (Smith et al., 2002; Authier et al., 2020) and includes the accepted study sensitivity and ethics for use of nonrodent species (ICH S7A Safety Pharmacology Studies for Human Pharmaceuticals, 2001; Smith et al., 2002; Authier et al., 2020). The study consisted of four acquisition sessions using a crossover design with dosing intervals of ≥ 48 h. In each experimental session, each dog received (*via* oral gavage) a different treatment: either vehicle or CPL500036 (1.5 mg/kg, 10 mg/kg or 30 mg/kg). Cardiovascular safety was assessed on arterial blood pressure (systolic, diastolic and mean), heart rate (HR) and electrocardiography (RR interval, PR interval, QRS duration, uncorrected QT and corrected QT (QTc) for heart rate using van de Water correction) acquired continuously for ≥ 2 h before dosing, and for ≥ 24 h post-dose. All parameters were evaluated before and at 13-time points after treatment (15 min, 30 min as well as 1 h, 2 h, 3 h, 4 h, 5 h, 6 h, 8 h,12 h, 16 h, 20 h, and 24 h after dosing).

### 2.14 Statistical analyses

Unless indicated otherwise, statistical analyses and data visualization were undertaken using Prism 9 (GraphPad, RRID:SCR_002798). The Shapiro–Wilk test was used to determine the normality of the data. Significance between the mean value of treatment groups was compared with the mean value of the control/vehicle group with one-way ANOVA with Dunnett’s correction. For the catalepsy test, data are presented as the latency to remove both forepaws from the bar, and were analyzed by mixed-design ANOVA (repeated factor: time, between factor and treatment) followed by Duncan’s *post hoc* test using Statistica (Tibco, Pala Alto, CA, United States, RRID:SCR_014213). Data presented as the area under the curve (AUC) were evaluated by one-way ANOVA followed by the Duncan or Holm-Sidak *post hoc* test. *p* < 0.05 was considered significant.

## 3 Results

### 3.1 IC_50_ and selectivity

**FIGURE 1:**
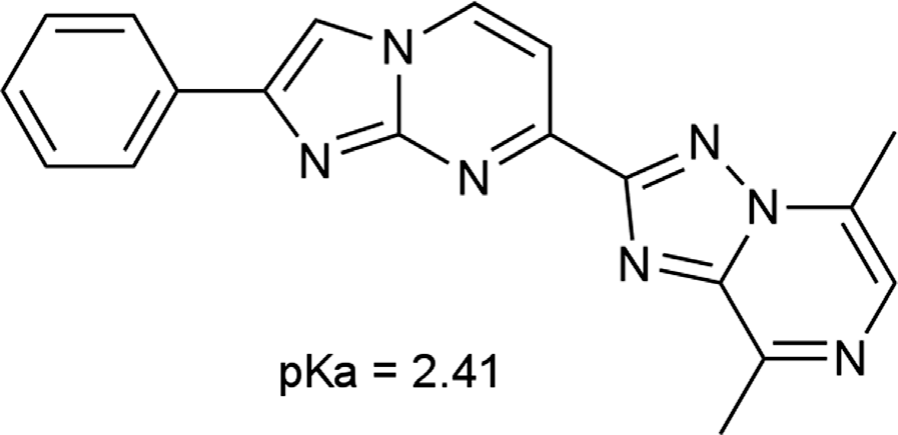
Chemical structure of CPL500036.

CPL500036 (Figure 1) was characterized by great potency against PDE10A (IC_50_ = 1 nM) as determined by a microfluidic mobility-shift assay. To assess selectivity, CPL500036 was tested at 1,000-fold higher concentration against 44 targets in a radioligand binding assay. The assay included screening against a panel of GPCRs, transporters, ion channels, nuclear receptor kinases and other non-kinase proteins, with which interaction may result in adverse effects (Bowes et al., 2012). Radioligand binding was not inhibited for any of the 44 protein targets by CPL500036 at 1,000 × IC_50_ (1 µM) (Table 1). An unusual result was detected for the muscarinic acetylcholine receptor M2 (antagonistic radioligand assay) because inhibition of the radioligand reached a negative value (−91.2%). The effect was investigated further in a functional assay and increasing concentrations of CPL500036 were studied in the presence of an agonist (acetylcholine) or antagonist (methoctramine) for the M2 receptor at concentrations corresponding to their EC_50_/IC_95_. CPL500036 induced a concentration-dependent inhibition of the control agonist response with an IC_50_ of 9.2 µM (Supplementary Figure S1)

**TABLE 1:** Selectivity of CPL500036 (1 μM). This table presents percent inhibition of specific binding of the control. “Interaction” is denoted by control specific binding > 25%.

Protein	% Inh	Protein	% Inh
adenosine A2A receptor (h)*	−8.1	µ -type opioid receptor (h)*	−1.1
alpha-1A adrenergic receptor (h)	1.8	serotonin receptor 1A (h)*	5.4
alpha-2A adrenergic receptor (h)	0.4	serotonin receptor 1B (r)	−7.3
beta-1 adrenergic receptor (h)*	1.1	serotonin receptor 2A (h)*	−8.6
beta-2 adrenergic receptor (h)*	8.2	serotonin receptor 2B (h)*	10.6
benzodiazepine receptor (GABAA) (r)*	−27.7	serotonin receptor 3 (h)	−5.7
cannabinoid receptor type 1 (h)*	8.8	glucocorticoid receptor (h)*	−3.2
cannabinoid receptor type 2 (h)*	−14.6	androgen receptor (h)*	5.3
cholecystokinin A receptor (h)*	−8.2	vasopressin receptor (h)*	−0.8
dopamine receptor type 1 (h)	−8.2	calcium channel, voltage-dependent, L type (r)	−7.2
dopamine receptor type 2 (h)*	−14.4	potassium voltage-gated channel subfamily H member 2 (h)	−19.6
Endothelin receptor (h)*	−16.8	α-dendrotoxin sensitive potassium channel (r)	−10.2
N-Methyl-d-aspartic acid receptor (r)	4.4	voltage-gated sodium channels site 2 (r)	−1.3
histamine receptor type 1 (h)	−14.6	norepinephrine transporter (h)	−14.5
histamine receptor type 2 (h)	1.8	dopamine transporter (h)	−6.9
Monoamine oxidase A (r)	1.0	5-HT transporter (h)	-9.5
muscarinic acetylcholine receptor type 1 (h)	−19.7	Cyclooxygenase 1 (h)	−9.5
muscarinic acetylcholine receptor type 2 (h)	−91.2	Cyclooxygenase 2 (h)	−0.4
muscarinic acetylcholine receptor type 3 (h)	0.7	phosphodiesterase 3A (h)	−15.2
alpha 4/beta 2 nicotinic acetylcholine receptor, (neuronal) (h)*	−6.1	phosphodiesterase 4 (h)	0.8
δ-type opioid receptor ẟ2 (h)*	6.9	lymphocyte-specific protein tyrosine kinase (h)	−1.9
κ-type opioid receptor (r)*	7.7	Acetylcholinesterase (h)	13.7

Abbreviations: h–human, r–rat, % inh. - % inhibition of control specific binding, * - agonist radioligand.

### 3.2 *In vitro* cardiovascular safety pharmacology

**FIGURE 2:**
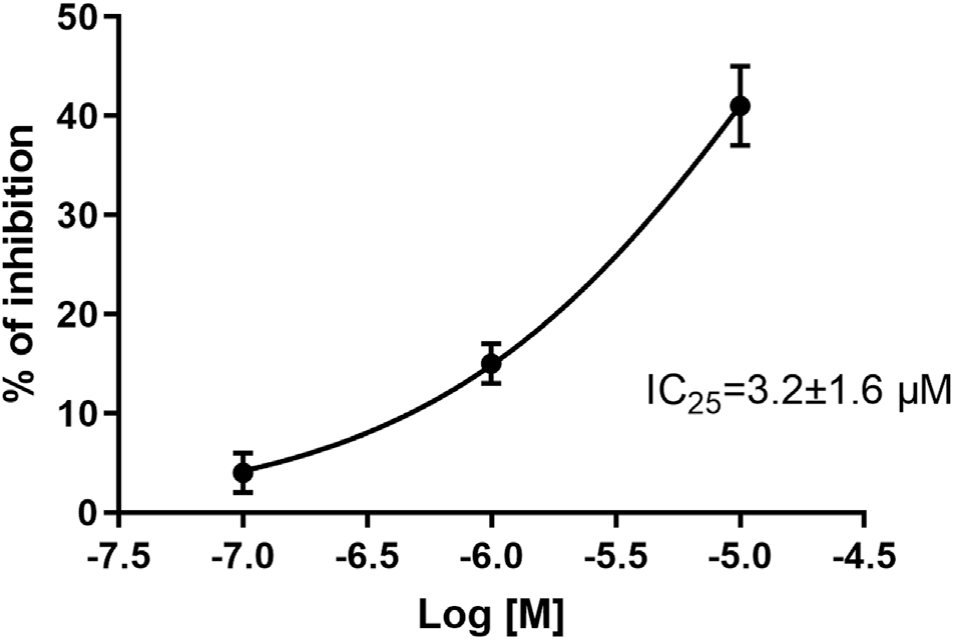
Effect of CPL500036 on inhibition of hERG tail current. CPL500036 was assayed in hERG-HEK-293 transfected cells using the manual patch-clamp method. The calculated IC_25_ was 3.2 μM ± 1.6 μM. Data are the mean ± SD (*n* = 3).

Evaluation of CPL5000036 effects on the cardiovascular system was undertaken in a series of dedicated experiments. An hERG *in vitro* assay was employed to evaluate the arrhythmic potential using a manual-clamp method in a whole-cell configuration. CPL500036 induced a concentration-dependent inhibition of hERG tail current with an IC_25_ of 3.2 μM ± 1.6 μM (Figure 2).

Cardiovascular safety was studied further in a human model using 3D cardiac spheroids. After 4 days of treatment with CPL500036 (0.0457 μM–100 µM), no effect on the number or size of spheroids, intracellular calcium concentration, mitochondrial mass, membrane potential or intracellular ATP was observed. However, CPL500036 impaired DNA structure with an IC_50_ of 37.4 µM, which indicated chromosomal instability and DNA fragmentation in these cells.

### 3.3 Cytotoxicity and genotoxicity *in vitro*

**FIGURE 3:**
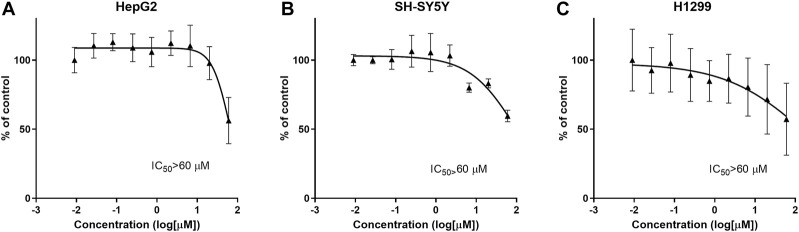
CPL500036 does not induce cytotoxic effects at ≤ 60 μM in HepG2 **(A)**, SH-SY5Y **(B)**, H1299, and **(C)** cells. Cells were treated 72 h before the MTT assay. Data are the mean ± SD (*n* = 3).

General cytotoxicity was studied in normal cells and cancer cells (SH-SY5Y [human neuroblastoma], HepG2 [human hepatocellular carcinoma] and H1299 [human non-small-cell lung cancer] *in vitro*. CPL500036 induced cell toxicity at IC_50_ < 60 μM in none of the cell lines tested (Figure 3).

Genotoxicity was tested in a concentration range of 0.025 mg/ml–25 mg/ml. Mutagenicity was evaluated in histidine-requiring auxotroph strains of *Salmonella typhimurium* (TA98, TA100, TA1535, and TA1537) and the tryptophan-requiring auxotroph strain of *Escherichia coli* (WP2 uvrA) in the presence and absence of metabolizing enzymes. No mutagenic activity of CPL500036 was recorded (data not shown).

### 3.4 Pharmacokinetic properties

To assess bioavailability and brain penetration, CPL500036 (3 mg/kg, p.o.) was administered to Wistar rats. Pharmacokinetic analyses (Table 2) indicated that CPL500036 had good bioavailability and penetrated the blood–brain barrier to result in good exposure to the brain (brain to plasma ratio = 0.49). The plasma protein binding is for rats is 92% ± 0.2%.

**TABLE 2:** Pharmacokinetic parameters after oral gavage of CPL500036 in a dose of 3 mg/kg in rats and 1.5 mg/kg in dogs’. Data are the mean ± SD.

	Rat (*n* = 5/timepoint)		Dog (*n* = 4)
Parameter	Plasma	Brain	Plasma
C_max_ (ng/ml)	237.8 ± 98.1	94.3 ± 28.5	98.3 ± 55.4
T_max_ (h)	5.8 ± 1.6	6.8 ± 3.3	1.3 ± 0.5
AUC_(0–24h)_ (ng*h/ml)	1,618.0 ± 298.3	843.6 ± 202.2	199.4 ± 105.5
t_1/2_ (h)	6.4 ± 6.2	–	2.1 ± 1.7
B/P	–	0.49 ± 0.06	–

Abbreviations: C_max_, maximum concentration; T_max_, time to C_max_; AUC_0–t_, area under the concentration–time curve; t_1/2_ - half-life of elimination, B/P, brain to plasma ratio.

### 3.5 Catalepsy

**FIGURE 4:**
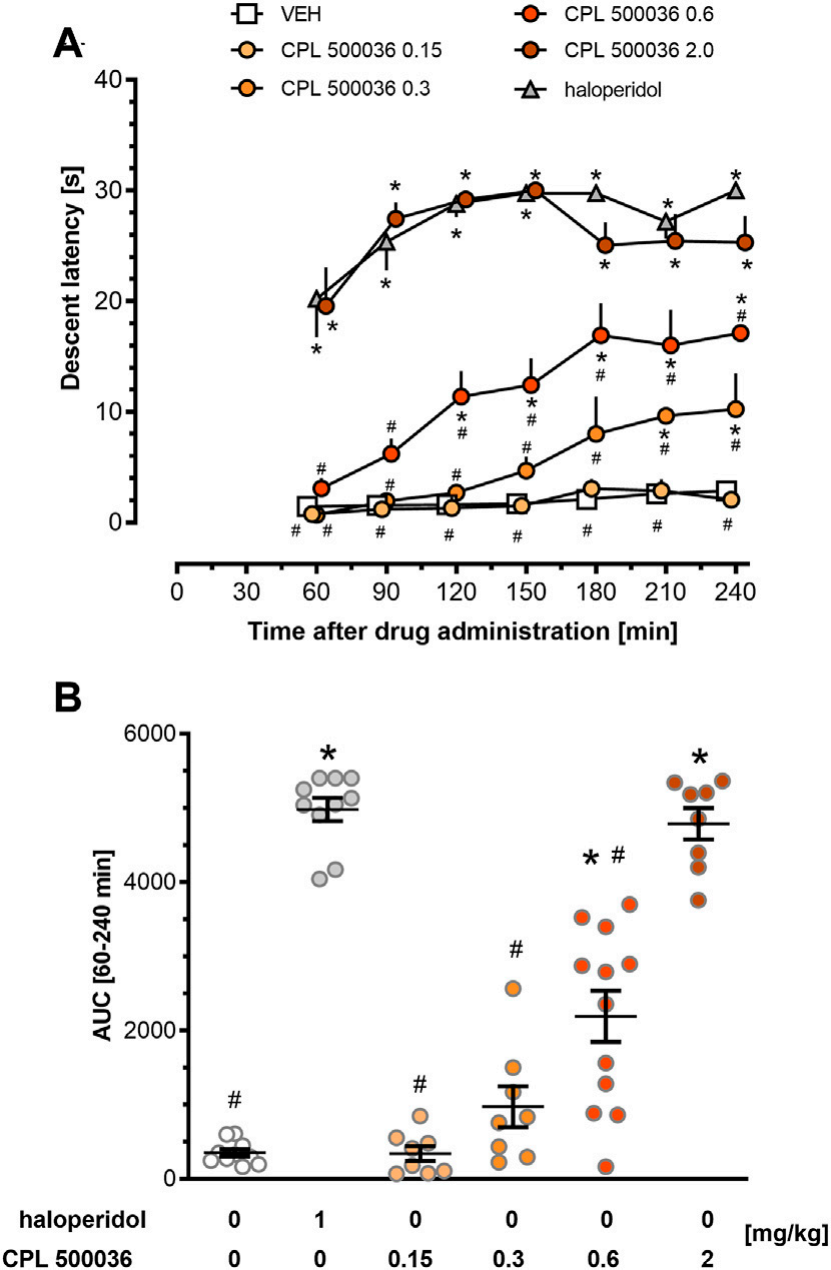
CPL500036 induces catalepsy at a dose of 0.6 mg/kg. The cataleptic response was assessed 60 min–240 min after administration of CPL500036 (0.15 mg/kg, 0.3 mg/kg, 0.6 mg/kg, and 2 mg/kg, p.o.) or haloperidol (1 mg/kg, i.p.). Catalepsy in the time course **(A)** and as the area under the curve **(B)** is presented as the mean and SEM. For the time-course, two-way mixed-design ANOVA demonstrated significant effects of an interaction between treatment and time: F (30, 300) = 3.19; *p* < 0.05; the *post hoc* test revealed differences between treatment and vehicle (*p < 0.05) and between treatment and haloperidol (#*p* < 0.05). One-way ANOVA on AUC data (lB) showed differences among groups: F (5,50) = 80.53; *p* < 0.05; the *post hoc* test demonstrated differences between treatment and vehicle *p < 0.05 and treatment and haloperidol effects #*p* < 0.05. *N* = 8–12/group.

Excessive activation of the dopamine D_2_ receptor and, subsequently, of the indirect pathway, can trigger extrapyramidal side-effects. Therefore, the cataleptogenic potential of CPL500036 employing the bar test was assessed in rats. CPL500036 (0.15 mg/kg, p.o.) did not induce catalepsy (Figure 4A). Administration of CPL500036 (0.3 mg/kg) resulted in a low cataleptogenic effect, and appeared only at 210 min and 240 min following administration. The catalepsy induced by a CPL500036 dose of 0.6 mg/kg was apparent 120 min–240 min following administration, but did not achieve the level elicited by haloperidol (1 mg/kg, i.p.). A CPL500036 dose of 2 mg/kg produced a strong cataleptogenic effect comparable with that of haloperidol (1 mg/kg, i.p.). AUC data (calculated from the duration of catalepsy) showed that the minimum effective dose (MED) of CPL500036 needed to elicit catalepsy was 0.6 mg/kg (Figure 4B).

### 3.6 Hyperprolactinemia

**FIGURE 5:**
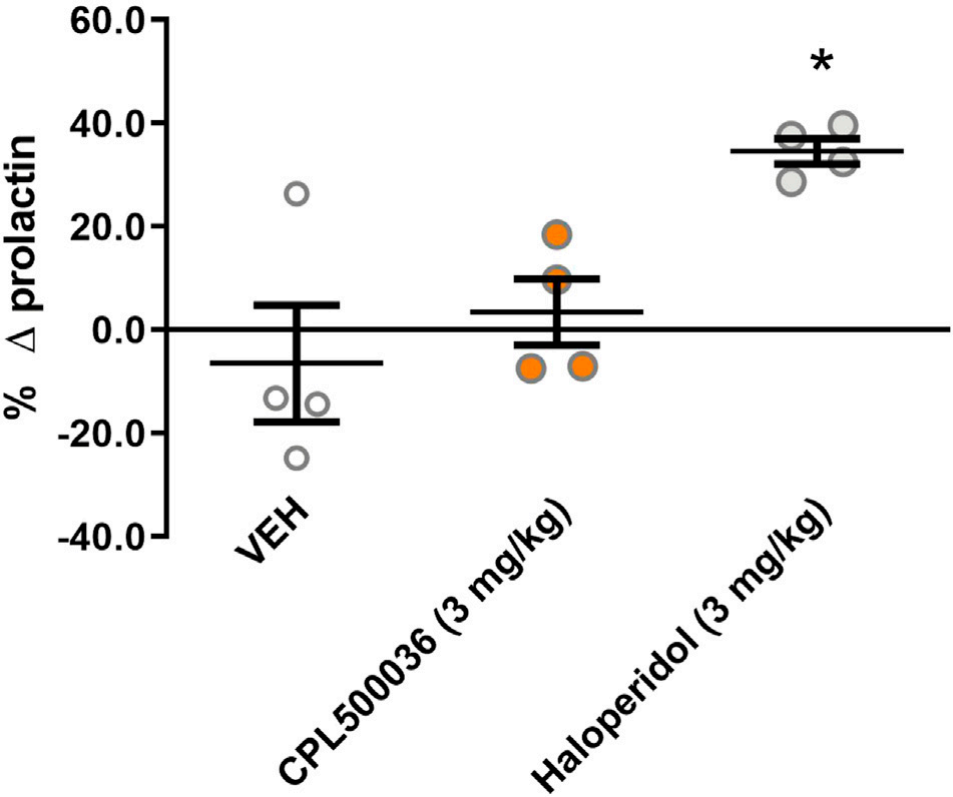
CPL500036 does not influence the prolactin level in rats. Animals were given (p.o.) vehicle, CPL500036 (3 mg/kg) or haloperidol (3 mg/kg). The results represent the relative change in the prolactin level before and 2 h after administration of the compound. Data are the mean ± SD. *p < 0.05 vs*.* vehicle group by Dunn’s *post hoc* test; *n* = 4.

Blockade of dopamine D2 receptors in the tuberoinfundibular dopamine pathway causes prolactin release and can lead to hyperprolactinemia if antagonists of dopamine D2 receptors are used. To investigate if the mode of action of CPL500036 affected the blood concentration of prolactin, the prolactin level was measured in rats before and 2 h after treatment with CPL500036 or haloperidol (positive control). Haloperidol induced significant prolactin release, in contrast to CPL500036 (3 mg/kg), which did not alter prolactin release (Figure 5).

### 3.7 Hyperglycemia

**FIGURE 6:**
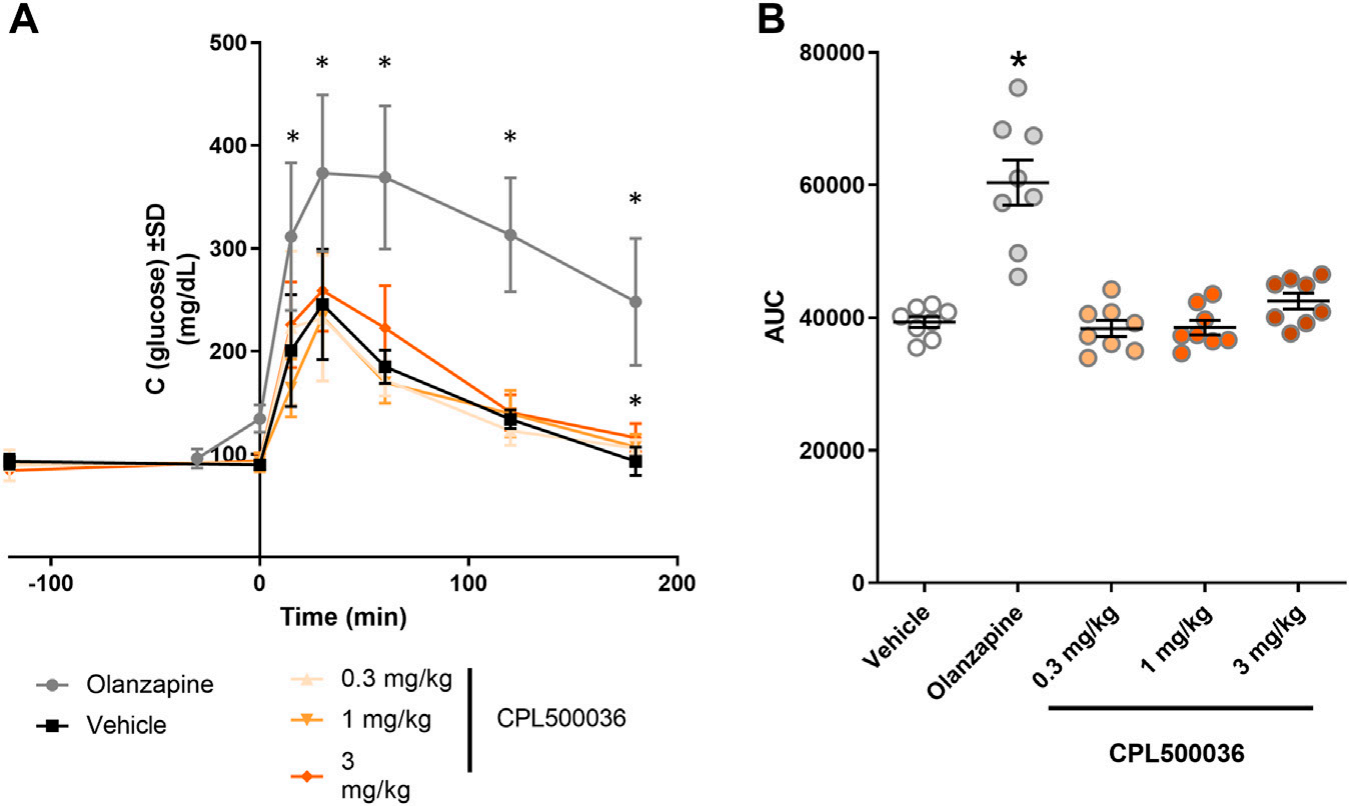
CPL500036 has no effect on the glucose level. The glucose-tolerance test was carried out 2 h after administration of CPL500036 (0.3 mg/kg, 1 mg/kg, and 3 mg/kg, p.o.) or olanzapine (10 mg/kg, s.c.). The glucose concentration in blood in the course of time **(A)** and as the area under the curve **(B)** is presented. Data are the mean ± SD. *p < 0.05 vs. vehicle group by Dunn’s *post hoc* test; *n* = 8.

Antipsychotics (particularly second-generation antipsychotics) are associated with metabolic adverse effects, including weight gain, type-2 diabetes mellitus and increase in the plasma glucose level in humans and rodents (Assié et al., 2008; Grajales et al., 2019). To assess the potential hyperglycemic effect of CPL500036, the glucose tolerance testwas carried out in rats. Administration of CPL500036 (≤ 3 mg/kg) did not elicit differences in glucose metabolism 3 h after giving a glucose bolus compared with after olanzapine administration (Figure 6).

### 3.8 *In vivo* cardiovascular safety

**FIGURE 7:**
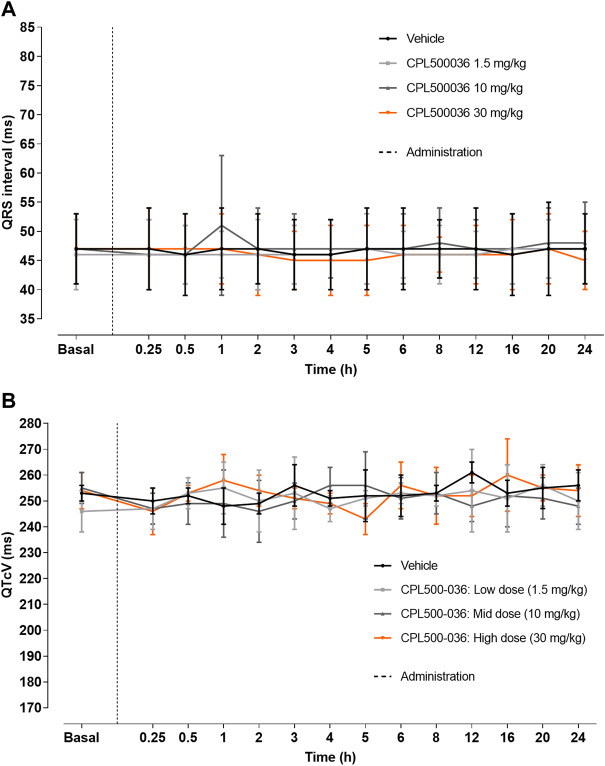
CPL500036 does not elicit changes in electrocardiography parameters. A single oral administration to Beagle dogs (1.5 mg/kg, 10 mg/kg or 30 mg/kg) did not induce alterations in QRS **(A)** or QTcV **(B)** intervals. Results are the mean ± SD; *n* = 4.

The potential for cardiovascular adverse effects was investigated further *in vivo*. CPL500036 (1.5 mg/kg, 10 mg/kg or 30 mg/kg, p.o.) was administered to Beagle dogs and blood pressure and electrocardiographic parameters (especially QT interval) were measured as a part of a safety-monitoring program. Remarkable variations in systolic, diastolic and mean blood pressures were not recorded after administration of vehicle or CPL500036 at any of the dose levels tested (data not shown). Noticeable alterations in any of the electrocardiography parameters analyzed (particularly QTcv and QRS duration) were not observed after administration of vehicle or CPL500036, regardless of the dose tested (Figure 7; Supplementary Figures S3–S9). Heart-rate alterations were detected after administration of vehicle or CPL500036. For each of the treatment groups, significant differences were not seen compared with the vehicle control group at any of the time points evaluated.

## 4 Discussion

The activity, selectivity, pharmacokinetics and safety of the novel PDE10A inhibitor CPL500036 that is under clinical development for treatment of basal-ganglia disorders were assessed. CPL500036 is characterized by great potency against PDE10A with an IC_50_ of 1 nM that is comparable with that of the most studied PDE10A inhibitors: MP-10 (0.18 nM), TP-10 (0.3 nM) and TAK-063 (0.3 nM) (Schmidt et al., 2008; Grauer et al., 2009; Harada et al., 2015). As shown before CPL500036 didn’t inhibit other PDE family members at a concentration of 100x IC50 (Moszczyński-Pętkowski et al., 2018). Moreover, CPL500036 presents a highly selective profile against further 42 proteins that may be involved in the most common adverse effects of potential drugs (Bowes et al., 2012). The exception was the result for the M2 muscarinic receptor. It can be asked why an increase in radioactive binding can occur. The negative inhibition may be interpreted as a non-specific effect linked to the physicochemical properties of the compound. In such a case precipitate formation between the compound and any assay components may lead to an increase in radioactivity. Such an effect may result in radioactivity that translates as a negative inhibition. However, such non-specific behavior usually is not limited to only one target. Another explanation may be receptor-specific and rely on an allosteric interaction that leads to increased binding of a radiolabeled ligand. As the latter option was more likely, a functional assay was employed to characterize the interaction in detail. CPL500036 didn’t trigger any agonist-like behavior but induced a concentration-dependent inhibition of the control agonist response in the antagonistic functional assay with an IC_50_ of 9.2 µM (Supplementary Figure S1). The results revealed a potential negative allosteric modulation of M2 receptor by CPL500036. This interaction is a potential safety risk because modulation of M2 receptors expressed in the central nervous system and cardiovascular system may lead to thermoregulation problems and tachycardia or increased cardiac output, respectively (Scarr, 2012). However, due to the relatively high IC_50_ for negative modulation, the risk of cardiac side-effects is low, but it was monitored closely in a set of follow-up cardiovascular experiments.

Neuropsychiatric drugs interact with the hERG channel, which results in a delay of ventricular repolarization and prolongation of the QT interval, thereby leading to potentially lethal Torsade de Pointes (Gintant et al., 2016). The likelihood of interaction with hERG increases if the molecule is a lipophilic basic amine without negatively ionizable groups or oxygen H-bond acceptors, which are the hallmarks of CPL500036 (Moszczyński-Pętkowski et al., 2018). Therefore, nonclinical assessment of blockade of hERG channels enables early exclusion of a highly undesirable interaction. CPL500036 induced a concentration-dependent inhibition of hERG tail current with an IC_25_ of 3.2 μM which exceeded, by > 3,000-times, the IC_50_ for PDE10A.

CPL500036 was investigated further in spontaneously beating cardiac 3D spheroids (which comprise cardiomyocytes and fibroblasts derived from human pluripotent cells). By assessing several parameters, the co-culture enabled prediction of potential hypertrophy or cardiotoxicity effects. CPL500036 (≤ 100 µM) did not influence most vital parameters (eg arrhythmia or myocardial damage), but it changed the DNA structure at AC_50_ = 37.4 µM. As stated above, the concentration was much higher than the expected therapeutic concentration range, but DNA damage was explored further by cytotoxicity and genotoxicity studies. To confirm these results and exclude further potential risks from the cardiovascular system, an *in vivo* safety assessment in conscious, telemetry-implanted dogs was undertaken. CPL500036 had no influence upon blood pressure or electrocardiographic parameters (especially QT interval and heart rate) in doses up to 30 mg/kg. Unfortunately, we didn’t have the opportunity to measure the concentration of CPL500036 in the heart of rats or dogs. Hence, CPL500036 did not elicit severe cardiovascular adverse effects in a preclinical assessment. Nevertheless, use of PDE10 inhibitors may have additional therapeutic advantages as PDE10A is involved into the pathology of cardiovascular diseases: heart failure and pulmonary arterial hypertension. PDE10A in both disorders modulates the pathological tissue remodeling and use of PDE10A inhibitors attenuated the symptoms *in vivo* (Tian et al., 2011; Huang et al., 2019; Chen et al., 2020). Taking into account the greater susceptibility for of cardiovascular diseases and heart failure in patients suffering from schizophrenia, use of CPL500036 may have additional benefits in controlling the other disease states.

Cytotoxicity was assessed in cell lines of neuronal, cancer or fibroblast origins. CPL500036 was non-cytotoxic at ≤ 60 µM. Based on the DNA damage observed in the 3D cardiac spheroid test and structural similarity to the DNA-binding agent 4′,6-diamidino-2-phenylindole, the potential risk of an undesired interaction with DNA emerged. Thus, genotoxic and mutagenic potential were investigated in the Ames test: CPL500036 presented no mutagenic potential.

Antipsychotic use is associated with several side-effects that limit the compliance of patients and influence their quality of life. In particular, extrapyramidal side-effects, hyperprolactinemia (common for first-generation antipsychotics) and metabolic complications caused by increases in the glucose level (more common among second-generation neuroleptics) are common concerns for these classes of molecules. These adverse effects were assessed in a set of *in vivo* experiments after showing that CPL500036 is bioavailable and penetrates the brain–blood-barrier in a highly efficient manner.

The extrapyramidal effects are a consequence of significant antagonism of dopamine D2 receptors in the striatum, which leads to a lack of movement initiation. Catalepsy is an accepted model for the prediction of extrapyramidal side-effects (Hoffman and Donovan, 1995). CPL500036 induced dose-dependent catalepsy with a MED of 0.6 mg/kg, with the maximal effect being observed at a dose of 2 mg/kg, and this effect was similar to that observed for haloperidol administered at a dose of 1 mg/kg. CPL500036 appeared to present a strong, dose-dependent cataleptic effect, unlike other PDE10A inhibitors (e.g., TAK-063, PDM-042, JNJ-42314,415) for which the effect was weaker than that for marketed antipsychotics and did not increase with increasing dose (Megens A. A. H. P. et al., 2014; Suzuki et al., 2015; Arakawa et al., 2016). We postulated that the attenuated catalepsy response observed for other PDE10A inhibitors is dependent upon activation of medium spiny neurons expressing dopamine D1 receptors that counteract the action modulated by the indirect pathway (Megens A.a. H. P. et al., 2014; Suzuki et al., 2015; Arakawa et al., 2016). The results obtained for CPL500036 could indicate less pronounced activation of the direct pathway, which may lead to a less “damped” antipsychotic effects mediated by neurons expressing dopamine D2 receptors. Additional studies clarifying this phenomenon and determining the therapeutic index are needed.

Second-generation antipsychotics impair insulin signaling and glucose uptake in many organs through interaction with on- and off-target proteins (Grajales et al., 2019). As a consequence, patients suffering from schizophrenia treated with antipsychotics may develop a metabolic syndrome that leads to considerably reduced compliance to medication regimens. CPL500036 represents a new class of antipsychotic agent that has no influence on glucose metabolism in doses up to 3 mg/kg. Hyperprolactinemia is a result of interactions with dopamine D2 receptors. Neurons of the tuberoinfundibular pathway do not express PDE10A, so CPL500036 (3 mg/kg) does not influence the acute prolactin level. The lack of effects on metabolism and prolactin release is in concordance with results for other PDE10A inhibitors that also do not influence either factor (Megens A. A. H. P. et al., 2014; Arakawa et al., 2016; Suzuki and Kimura, 2018).

Based on the plasma protein binding and pharmacokinetic data, the free concentration of CPL500036 in rats was determined to be 5% so the unbound fraction of the compound was estimated to 27.0 nM (11.9 ng/ml) and 10.7 nM (4.7 ng/g) at C_max_ for plasma and brain, respectively. Moreover, no active metabolites were identified in a hepatocyte stability assay (data not shown, *n* = 6). All described studies covered concentrations that exceeded the CPL500036 minimal activity of 1 nM, However, assuming a linear increase in exposition, the MED (Minimum Effective Dose) for catalepsy of 0.6 mg/kg would correspond to 2 nM concentration of CPL500036 in the brain at the t_max_ being fairly at the limit of determined IC_50_. Yet, CPL500036 showed therapeutic activity already at a dose of 0.1 mg/kg in Parkinson’s disease (Lenda et al., 2021) and psychosis animal models (manuscript in preparation). Moreover, the studies on other PDE10A inhibitors showed that the therapeutic effect of PDE10A was achieved at the enzyme occupancy of about 30%, suggesting that the use of doses leading to a free fraction concentration near IC_50_ was effective (Tomimatsu et al., 2016; Delnomdedieu et al., 2017). Moreover, CPL500036 is bioavailable in human and generally safe and well tolerated with no serious adverse effects (Janowska et al., 2019, manuscript in preparation).

## 5 Conclusion

CPL500036 is a highly active, selective, orally bioavailable and brain-penetrable novel PDE10A inhibitor. As a potential antipsychotic drug, its safety profile is distinct from that of approved antipsychotics that induce hyperprolactinemia or hyperglycemia. We demonstrated that the risk for genotoxic or cardiovascular side-effects was very low. CPL500036 may be explored further and its safety profile justifies its clinical development.

## Data Availability

The raw data supporting the conclusion of this article will be made available by the authors, without undue reservation.
