# Supplementary material for: A PDE10A inhibitor CPL500036 is a novel agent modulating striatal function devoid of most neuroleptic side-effects
Source: Front Pharmacol. 2022 Nov 9;13:999685. doi: 10.3389/fphar.2022.999685 (PMC9681820; doi:10.3389/fphar.2022.999685)
Supplement: Supplementary file 1 [file DataSheet1.PDF]

Supplementary Material

Supplementary Table 1 Positive controls **-** mitomycin C and dasatinib – induced several changes in safety parameters in 3D cardiac spheroids.

	**Dasatinib**		**Mitomycin C**	
**Parameter**	**MEC (µM)**	**AC_{50} (µM)**	**MEC (µM)**	**AC_{50} (µM)**
Spheroid count	NR	NR	2	NR
Spheroid size	0.469	23.4	2.97	>100
DNA structure	2.92	>50	0.704	4.41
Calcium	43.0	>50	79.1	>100
Mitochondrial mass	2.43	>5	<0.04	0.731
Mitochondrial membrane potential	16.82	26.4	1.89	3.88
Cellular ATP	0.075	0.374	0.243	0.766

Abbrevations: MEC - Minimum effective concentration that significantly crosses vehicle control threshold; AC_{50} - The concentration at which 50% maximum effect is observed for each cell health parameter; **NR** - No response observed.

Supplementary Table 2 Results for positive controls and CPL5000036 for the genotoxicity study (n=3)

	**Without metabolic activation (-S9 mix)**		**With metabolic activation (+S9 mix)**	
**Comppund**	**Revertants Mean±SD**	**Mutation rate**	**Revertants Mean±SD**	**Mutation rate**
Salmonella typhimurium TA98				
CPL500036 (5000µg/plate)	14.3±1.15	0.74	18.3±8.02	0.92
NPD (4µg/plate) (-S9 mix)<br>2AA (2µg/plate) (+S9 mix)	335.0±57.51	18.6	1682.7±156.02	84.13
Salmonella typhimurium TA100				
CPL500036 (5000µg/plate)	66.3±1.08	1.02	62.3±9.02	0.82
SAZ (2µg/plate) (-S9 mix)<br>2AA (2µg/plate) (+S9 mix)	1093.0±64.66	12.76	1464.0±361.88	19.26
Salmonella typhimurium TA1535				
CPL500036 (5000µg/plate)	12.0±5.57	1.13	8.3±5.13	0.6
SAZ (2µg/plate) (-S9 mix)<br>2AA (2µg/plate) (+S9 mix)	940.0±91.95	74.21	159.3±15.57	11.38
Salmonella typhimurium TA1537				
CPL500036 (5000µg/plate)	4.0±4.36	0.71	6.0±5.20	0.69
9AA (50µg/plate) (-S9 mix)<br>2AA (2µg/plate) (+S9 mix)	287.0±65.60	50.65	78.0±7.94	9.0
Escherichia coli WP2 *uvrA*				
CPL500036 (5000µg/plate)	22.3±2.08	1.12	42.3±2.89	1.76
MMS (2µg/plate) (-S9 mix)<br>2AA (2µg/plate) (+S9 mix)	1184.7±48.01	33.85	203.7±3.79	8.49

Abbrevations: NPD - 4-Nitro-1,2-phenylenediamine; SAZ – Sodium Azide; 9AA - 9-Aminoacridine; 2AA - 2-aminoanthracene; MMS - Methyl methanesulfonate

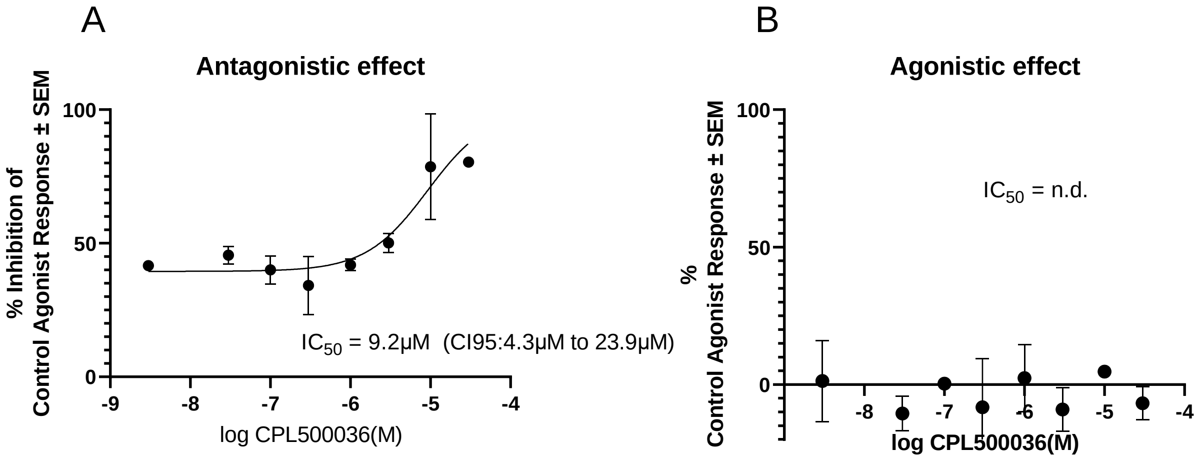

Supplementary fig. 1 Assesment of possible allosteric modulation in M2 muscarinic (human) receptor by CPL500036 in CHO cells. CPL500036 did not trigger any agonist-like behavior but induced a concentration-dependent inhibition of the control agonist response with IC50 equal to 9.2 µM

The study was perfomed by Eurofins Cerep (Poitiers, France) according to the service-provider's protocol. Briefly, CHO-K1 cells overexpressing M2 muscarinic receptor (human, XM_011515769) were treated with increasing concentration of CPL500036 (3nM-30µM) at a constant concentration of agonist (acetylocholine) or antagonist (methoctramine). To assess an agonistic and antagonistic allosteric effects, EC95 of the reference antagonist and EC50 of the reference agonist were used, respectively. The potential antagonistic allosteric effect was studied at the EC50 of the agonist instead of the EC95 to see if the compound increases or decreases the effect of the agonist. Cells were treated for 10 min with CPL500036. cAMP was detected by use of HTRF (Homogeneous Time Resolved Fluorescence) method.

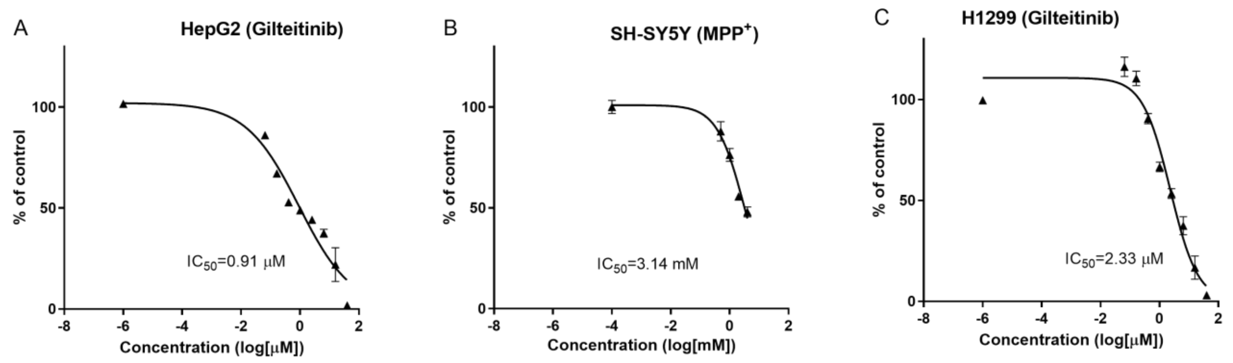

Supplementary fig. 2 Positive controls induced cytotoxicy in the tested cell lines. Cells were treated 72 h before the MTT assay. Data are the mean ± SD (n = 3).

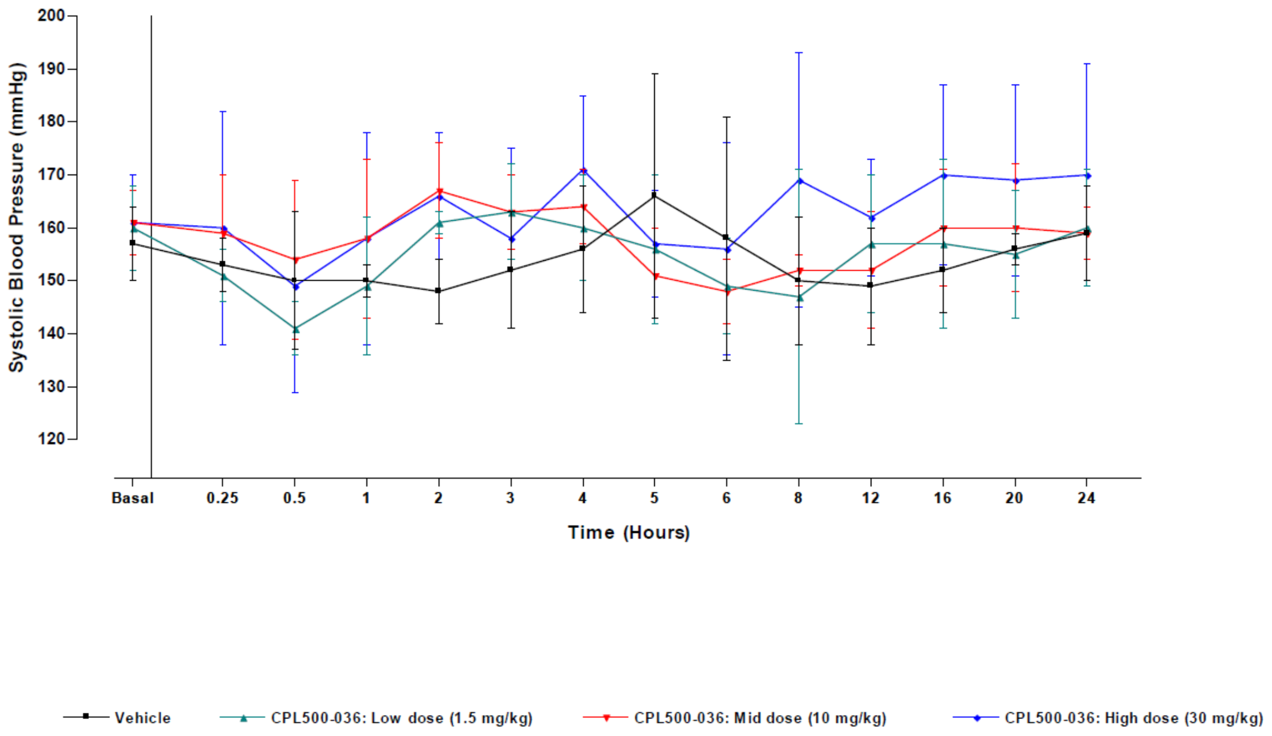

Supplementary fig. 3 A single oral administration to Beagle dogs (1.5, 10 or 30 mg/kg) did not induce alterations in Systolic Blood Pressure. Results are the mean ± SD; n = 4

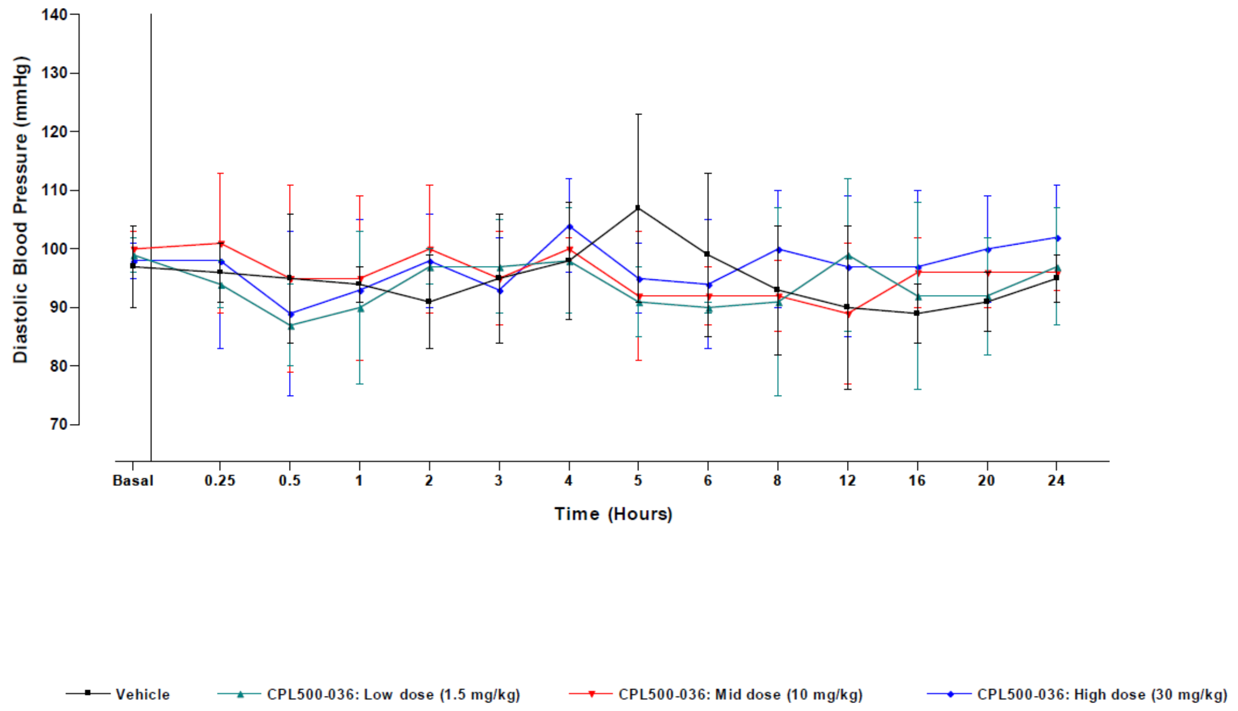

Supplementary fig. 4 A single oral administration to Beagle dogs (1.5, 10 or 30 mg/kg) did not induce alterations in Diastolic Blood Pressure. Results are the mean ± SD; n = 4

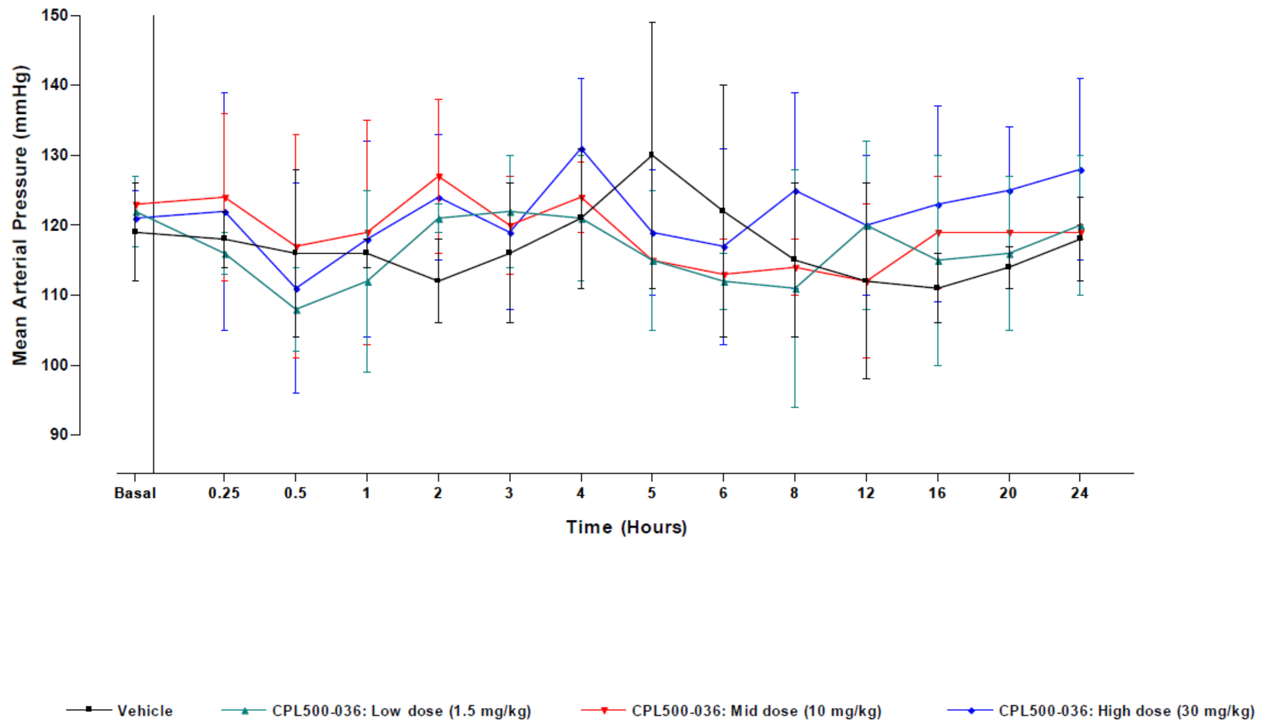

Supplementary fig. 5 A single oral administration to Beagle dogs (1.5, 10 or 30 mg/kg) did not induce alterations in Mean Blood Pressure. Results are the mean ± SD; n = 4

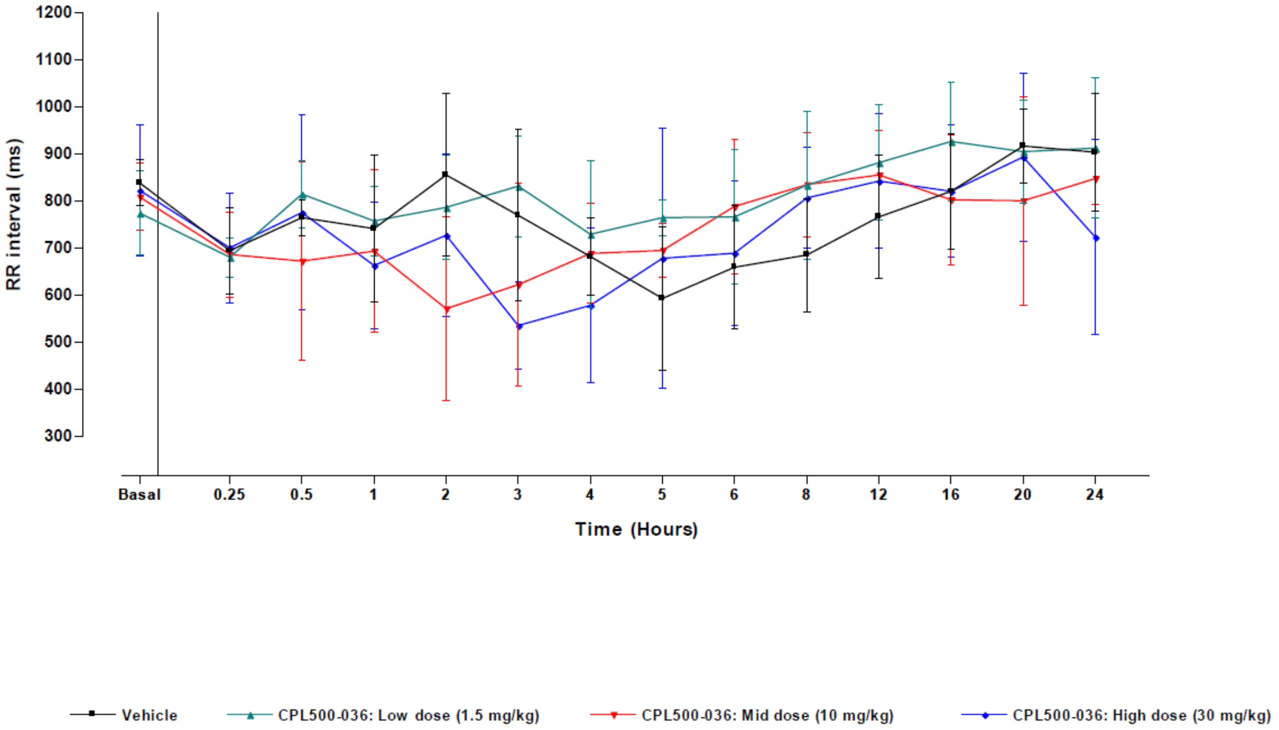

Supplementary fig. 6 A single oral administration to Beagle dogs (1.5, 10 or 30 mg/kg) did not induce alterations in RR interval. Results are the mean ± SD; n = 4

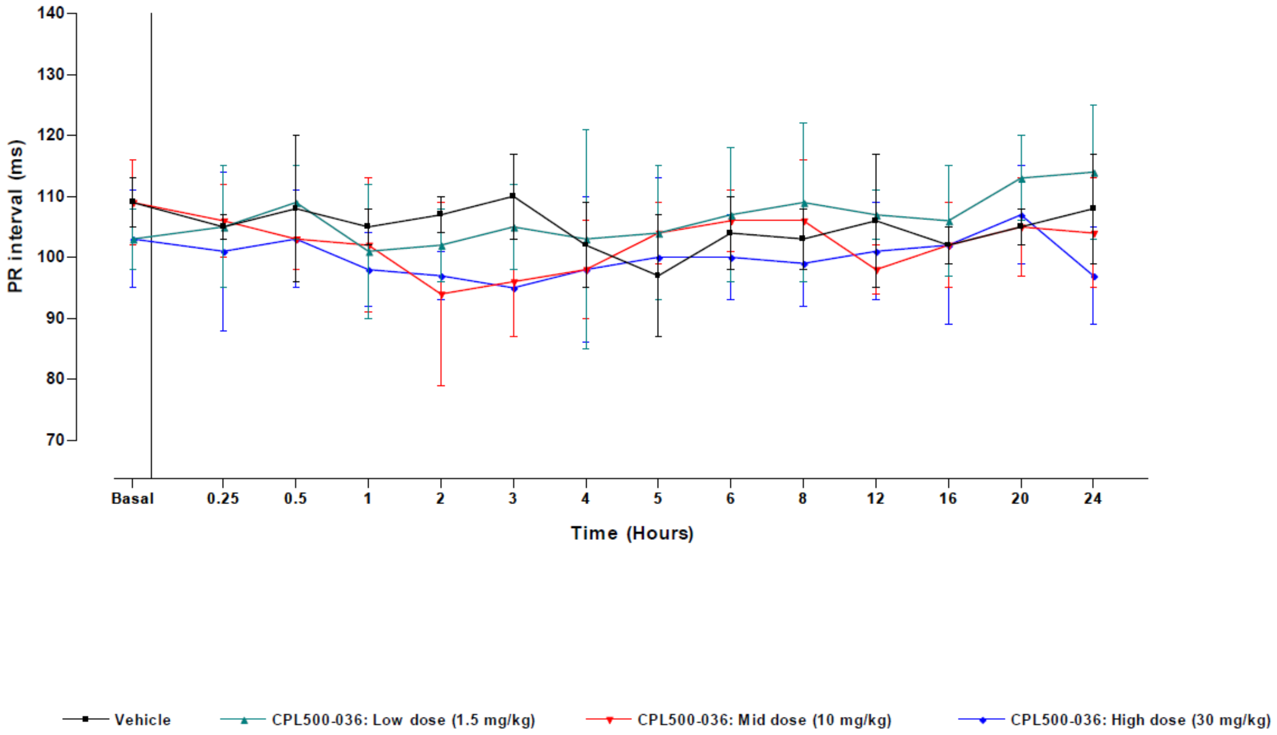

Supplementary fig. 7 A single oral administration to Beagle dogs (1.5, 10 or 30 mg/kg) did not induce alterations in PR interval. Results are the mean ± SD; n = 4

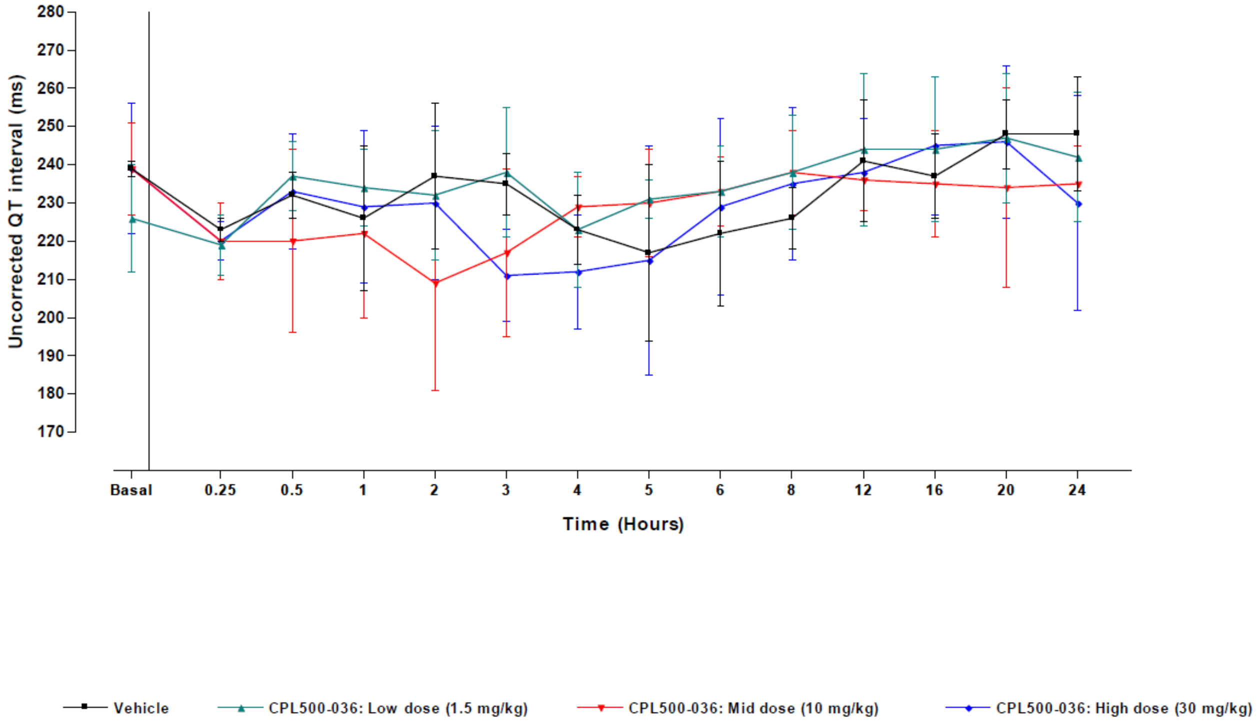

Supplementary fig. 8 A single oral administration to Beagle dogs (1.5, 10 or 30 mg/kg) did not induce alterations in uncorrected QT interval. Results are the mean ± SD; n = 4

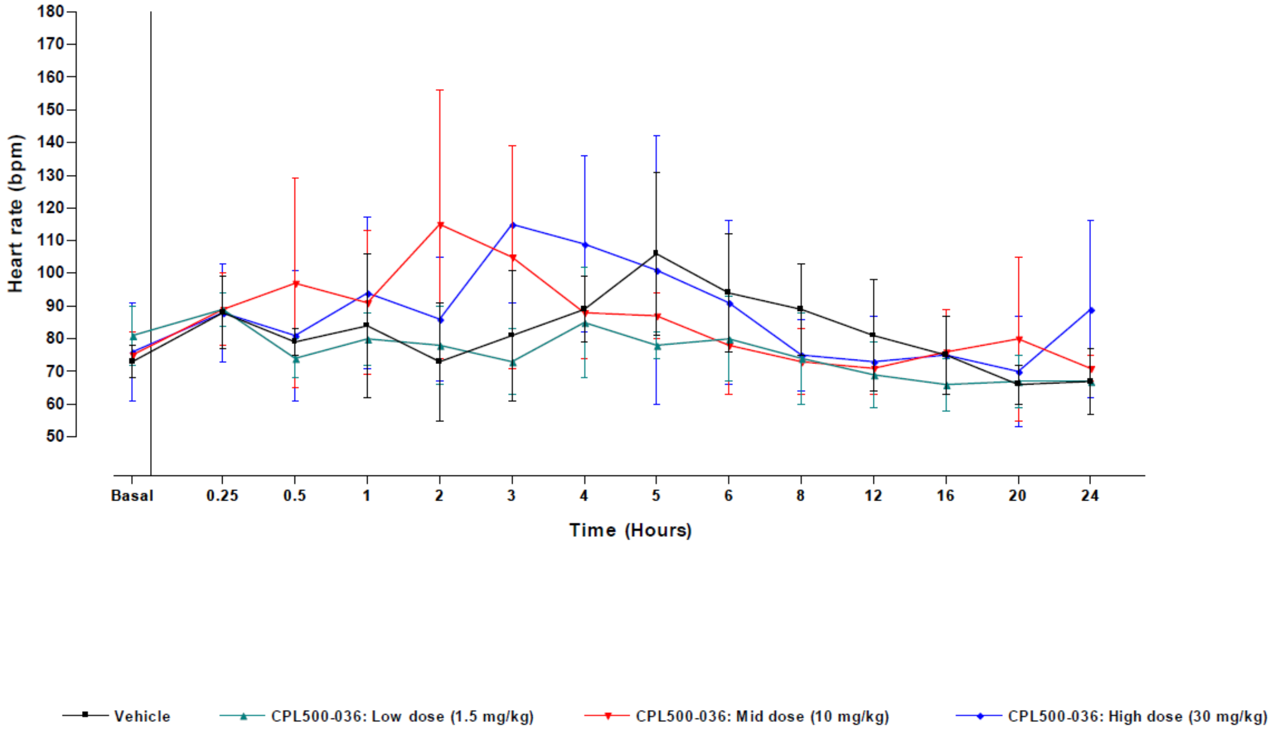

Supplementary fig. 9 A single oral administration to Beagle dogs (1.5, 10 or 30 mg/kg) did not induce alterations in heart rate. Results are the mean ± SD; n = 4
